# The relationship between intellectual curiosity and adolescents’ mathematical problem-solving ability: A moderated mediation model

**DOI:** 10.1371/journal.pone.0344349

**Published:** 2026-03-19

**Authors:** Yongzhao Wang, Lijun Zhou, Bingqing Xie, Lisha Wang, Hua Jin

**Affiliations:** 1 School of Mathematics and Statistics, Anyang Normal University, Anyang, China; 2 School of Management and Economics, North China University of Water Resources and Electric Power, Zhengzhou, China; 3 Department of Chinese Language and Literature, Gyeongsang National University, Jinju, Korea; University College London, UNITED KINGDOM OF GREAT BRITAIN AND NORTHERN IRELAND

## Abstract

The ability to solve mathematical problems is one of the key skills for students to excel in mathematics. Based on PISA 2022 data from Ireland (N = 5,521), the current study constructed a moderated mediation model to systematically examine how intellectual curiosity (IC) influences mathematical problem-solving ability (MPSA) through the mediation of self-efficacy (SE), and to test the moderating role of perseverance (PE). The results indicated significant positive correlations among IC, SE, PE, and MPSA. Further mediation analysis revealed that IC not only exerted a direct effect on MPSA, but also affected MPSA indirectly through the role of SE, accounting for 45.40% of the total effect. Moreover, the moderated mediation analysis uncovered a dual role of PE in the mechanism through which IC influences MPSA: it negatively moderated the paths from IC to SE and from IC to MPSA, while positively moderating the path from SE to MPSA. Multi-group analysis revealed significant heterogeneity in the mechanism across gender and economic, social, and cultural status (ESCS). Specifically, male students relied more heavily on the mediating path of SE, whereas female students exhibited a stronger direct effect of IC. Students with low ESCS primarily depended on the direct drive of IC, whereas their high-ESCS counterparts achieved ability enhancement more through the mediating pathway of SE. These findings elucidate the cross-group psychological mechanisms influencing MPSA, validate the applicability of self-determination theory and social cognitive theory in standardized educational contexts, provide empirical evidence for Ireland to formulate differentiated and targeted mathematics education intervention policies, and offer practical insights for promoting the comprehensive development of adolescents.

## Introduction

With the rapid advancement of technology, higher standards for talent cultivation have been set than in the past, positioning problem-solving ability as a key competency and essential skill for contemporary learners. The ability not only lays a solid foundation for learners’ adaptation and development in social life, but also serves as a crucial support for lifelong learning. Consequently, it has garnered widespread attention within the education sector, prompting numerous nations to incorporate it into their core educational frameworks [1,2]. Similarly, the Organisation for Economic Co-operation and Development (OECD) identifies problem-solving as a key 21st-century competency, incorporating its assessment in the Programme for the International Assessment of Adult Competencies (PIAAC) [3]. Mathematics, as a fundamental discipline, plays an extremely important role in modern society [4]. It constitutes a core instrument for scientific inquiry and technological innovation, while simultaneously serving as a critical pathway for cultivating logical reasoning, abstract thinking, and problem-solving competencies. Mathematical problem-solving ability (MPSA) constitutes a central component of mathematical literacy, significantly influencing students’ academic progression and career prospects. According to the Programme for International Student Assessment (PISA) 2022 framework, mathematical literacy is defined as an individual’s ability to reason mathematically and solve problems in varied real-world contexts through key cognitive processes including formulation, application, interpretation and evaluation, and reasoning [5]. However, the inherent complexity of mathematical learning presents significant challenges for many students in developing MPSA. Therefore, it is essential to systematically explore the multidimensional factors influencing students’ MPSA. Such exploration could meet the practical demands of national and global talent development strategies, also offer a vital means of enhancing mathematics teaching and learning outcomes.

The adolescent stage marks the onset of the “formal operational stage” in cognitive development, representing a pivotal transition from concrete imagery to abstract logical thinking. It also constitutes a critical juncture in the progression from primary education to secondary education or vocational pathways, rendering the period both distinctive and crucial [6]. By this stage, adolescents have developed foundational problem-solving skills that significantly influence their future academic and life outcomes. It is therefore unsurprising that the PISA targets 15-year-old students as its primary group [7]. Ireland, as an OECD member country, demonstrated a striking subject-specific performance disparity in the 2022 PISA cycle. While Irish students scored significantly above the OECD average in reading (516 points) and science (504 points), their performance in mathematical literacy (492 points) fell below the benchmark of 500 points [8]. The imbalance among subjects not only highlights potential shortcomings in Ireland’s education system in the field of mathematics, but also provides unique research value for exploring the mechanisms influencing MPSA within a relatively standardized curriculum framework. However, national-level overall performance may mask heterogeneity among different student groups. The existing research suggests that factors such as gender and economic, social and cultural status (ESCS) can influence learning experiences, social expectations and access to resources. As a result, there may be variations in the way non-cognitive factors influence MPSA [9]. Specifically, with regard to gender, the relevant studies have found that male students often report higher levels of self-efficacy in mathematics, and that their academic performance tends to be more dependent on the belief. In contrast, female students may depend more on sustained effort and perseverance for achievement. The phenomenon indicates the existence of potential gender-specific patterns in the operation of motivational and belief systems [10–12]. In terms of ESCS, students from higher socioeconomic backgrounds usually have access to richer educational resources and emotional support, which can boost their self-efficacy and help them to benefit more from non-cognitive factors. Conversely, students with fewer resources may need to rely more on motivation and perseverance to overcome environmental constraints [13–15]. Therefore, an analysis that focuses solely on the overall level risks obscuring the full picture of the mechanisms influencing MPSA and failing to provide precise targets for differentiated educational interventions. Based on the above discussion, the present study, focusing on Irish adolescents, aims to explore the key factors influencing their MPSA at the overall level and further examines the heterogeneity of the influencing mechanisms across groups through multi-group analysis. Given that PISA assesses 15-year-old students, who are highly homogeneous in age and grade level [16,17], the current study will concentrate on the two key dimensions of gender and ESCS to deeply explore the universality and specificity of the aforementioned mechanisms across different student groups. Such exploration not only provides a cross-group theoretical validation for international assessment systems, but also offers a solid empirical basis for formulating targeted mathematics education enhancement strategies for different student groups in Ireland and similar educational systems.

In the field of adolescents’ mathematics education, problem-solving ability is regarded as crucial for cultivating higher-order thinking and lifelong learning skills [18,19]. Contemporary educational psychology research indicates that focusing solely on cognitive skills is insufficient to explain ability differences among students, non-cognitive factors play essential roles, such as motivation, beliefs, and personality traits [20–22]. To systematically investigate the effects of these factors, the current study adopts an integrated perspective drawing on social cognitive theory (SCT) and self-determination theory (SDT), selecting three core representative variables. Intellectual curiosity (IC), as a prototypical form of intrinsic motivation from SDT, drives exploratory behaviors. Self-efficacy (SE), a core belief within SCT, determines confidence in facing challenges and the application of strategies. Perseverance (PE), a key volitional trait, ensures sustained effort in difficult situations. The three factors interact synergistically, collectively forming a key non-cognitive system influencing MPSA [23–25].

### The foundational role of intellectual curiosity as an intrinsic motivation in mathematical problem-solving ability

Among the numerous non-cognitive factors influencing students’ academic performance, motivation is regarded as the core driver that activates and sustains learning behaviors. IC, a key form of intrinsic motivation, is particularly crucial for the mathematical problem-solving process, which requires continuous exploration and cognitive effort [26]. IC, also referred to as epistemic curiosity, represents an individual’s inherent desire and pursuit of knowledge, defined as the “motivation to seek, engage in, enjoy, and pursue cognitive effort” [27,28]. It is not merely a transient emotional state but a stable psychological disposition that drives individuals to voluntarily engage in cognitively demanding activities and explore to fill knowledge gaps [26]. Within the Big Five personality model, IC is considered as a key facet of the “Openness to Experience” trait, closely associated with an individual’s intellectual engagement, learning interest, and exploratory behaviors [29,30]. SDT provides a crucial theoretical perspective for understanding the central role of IC. The theory posits that high-quality intrinsic motivation is fostered when an individual’s three basic psychological needs including autonomy (self-direction), competence (mastery of the environment), and relatedness (connection with others) are satisfied [23,31]. When students learn driven by IC, their need for autonomy is fully respected, as their exploratory behaviors are self-initiated rather than coerced. Furthermore, each instance of mastering knowledge during the process of exploring further fulfills their need for competence [32].

Extensive empirical research has validated the positive impact of IC on academic development [33–36]. However, these studies have certain methodological and developmental limitations. For example, Hardy et al. (2020) conducted a study involving 285 adult participants and revealed that IC indirectly enhances academic performance by improving creative problem-solving abilities. Yet, the limited sample size may have compromised statistical power [37]. Furthermore, conclusions drawn from the relatively stable cognitive and emotional developmental stage in adults require careful validation before they can be directly extended to adolescents who are undergoing pivotal phases of self-identity and cognitive style formation. Similarly, although Liu et al. (2024) employed a large sample size (N = 45,972) and confirmed that active learning pedagogies indirectly enhance problem-solving abilities by stimulating student curiosity, their study spanned the broad educational stages from primary to secondary school [27]. It failed to focus specifically on late adolescence, a period marked by rapid development in abstract thinking and self-concept. These methodological and developmental limitations highlight the importance of using PISA’s nationally representative, large-scale adolescent data to systematically examine the role of intellectual curiosity in developing mathematical problem-solving abilities in adolescents. Furthermore, Irish students exhibit a pronounced ’subject imbalance’ in PISA [8], a particularly thought-provoking phenomenon within a relatively standardized curriculum system. Furthermore, in the specific context of mathematics education, students with high IC exhibit greater exploratory tendencies. They are more inclined to delve actively into the essence of mathematical problems, proactively applying their acquired knowledge and strategies to find solutions [38–40]. In summary, as a core intrinsic motivation, IC is posited to promote the development of adolescents’ MPSA by driving deep learning engagement and strategic exploration.

### Self-Efficacy: The key cognitive bridge connecting motivation and ability

Within the psychological systems that shape academic achievement and development, SE, as an individual’s belief in and judgment of their own capabilities, plays a critical role. It serves not only as a key driver of learning behaviors, but also as a crucial psychological mechanism that translates intrinsic motivation into actual performance [41–44]. SE, originating from SCT, refers to an individual’s confidence in their ability to organise and execute the courses of action required to manage prospective situations in a specific domain [45]. The current study focuses specifically on mathematical self-efficacy, defined as students’ confidence in their capacity to successfully solve mathematical problems and complete mathematics learning tasks [46,47]. It should be particularly noted that, based on the operational definition derived from SCT, the SE examined in the current study fundamentally differs from the unfounded self-aggrandizement that may be perceived as “arrogance” in everyday life [48,49]. SE constitutes an individual’s subjective belief and judgment regarding their capacity to successfully accomplish tasks within specific domains. Its formation arises from the integration of multiple information sources, particularly mastery experiences (personal past successes and failures), vicarious experiences (observing others’ successes), verbal persuasion, and physiological and emotional states [22]. A high level of SE, well-calibrated to one’s actual capabilities, exerts positive effects by enhancing learning engagement, strategic application, and persistence in the face of difficulties [50]. The research by Vancouver et al. (2014) further indicates the need to distinguish between well-calibrated self-efficacy and unrealistic overconfidence [51]. The latter typically represents an exaggerated self-perception lacking reliable experiential support, potentially leading to inadequate preparation or misjudged risks. Consequently, the present research examines self-efficacy as a motivational, domain-specific belief system whose value lies in driving and sustaining goal-directed actions, rather than in objectively calibrating one’s capabilities.

According to SCT, a person’s beliefs have a profound influence on their choice of activities, the level of effort they exert, and their persistence in overcoming obstacles. Robust SE has multifaceted positive effects on students’ learning behaviors and achievement. The previous research indicates a significant positive correlation between SE and MPSA [50, 52-53]. Specifically, students with high SE are more likely to perceive complex mathematical problems as challenges to be overcome rather than threats. Consequently, they demonstrate a greater willingness to invest cognitive effort, employ flexible strategies and exhibit greater resilience, ultimately performing better in the process of problem solving [54,55]. Furthermore, as a core agentic belief, the formation and development of SE are influenced by other psychological factors. IC, as a powerful intrinsic motivation, drives students to actively explore mathematical knowledge. They accumulate positive cognitive experiences and minor successes during the process of encountering new problems, experimenting with strategies, and experiencing “aha” moments. These experiences are essential for constructing strong SE. Strong IC is thus likely to effectively shape and enhance students’ confidence in their mathematical abilities by providing them with continuous success validation [56,57]. However, the existing research evidence still leaves room for exploration in systematically integrating how IC influences competence by shaping SE, and caution is warranted when extrapolating these findings to adolescent populations. For instance, Chang et al. (2023) provided causal evidence for the enhancement of self-efficacy through joyful curiosity through a two-and-a-half-year longitudinal study. However, their sample comprised undergraduate and postgraduate students [58], whose self-concepts were relatively stable as adult individuals. Consequently, their findings warrant caution when generalized to adolescents undergoing critical self-identity exploration. Although Jones et al. (2023) employed a sufficiently large sample [59], their cross-sectional design precludes establishing causal relationships between variables. Furthermore, the study’s focus on medical students as a specific cohort limits the generalizability of its findings. Similarly, Liberman et al. (2019) exhibited notable developmental limitations due to their cross-sectional design and field-based adult sample [60]. These methodological and developmental limitations underscore the unique value of systematically examining the complete motivational-belief-ability pathway using nationally representative, large-scale adolescent data. In summary, existing theories separately highlight curiosity’s role as an intrinsic motivator driving exploratory behavior, and self-efficacy’s function as a core agency belief regulating learning engagement and performance. From an integrative perspective, IC likely serves as a crucial source for building robust mathematical SE by creating sustained, curiosity-driven mastery experiences for students. The belief of “I can do it” then translates into greater effort, better strategy use, and enhanced resilience when confronting complex mathematical problems, ultimately improving problem-solving performance. The IC → SE → MPSA transmission pathway aligns with the SCT framework of interaction between personal factors (beliefs), behavior, and environment. SE serves as the pivotal personal agency element, translating intrinsic motivation into sustained cognitive and behavioral engagement [22]. Therefore, from an integrated perspective of SDT and SCT, SE likely serves as a pivotal bridge between adolescents’ IC and their MPSA. It functions not only as a crucial transformative hub between motivation and competence but also as a core psychological mechanism for understanding how non-cognitive factors synergistically influence mathematical academic performance.

### The synergistic role of perseverance: a personality safeguard for maintaining and strengthening the motivational pathway

Among the numerous factors shaping students’ academic performance, stable personality traits are key predictors of long-term success. PE, as a trait characterised by sustained effort and enthusiasm in the pursuit of goals, is especially crucial in the problem-solving process of mathematics, which demands overcoming numerous difficulties. Accordingly, PE has consistently emerged as a significant predictor of mathematical problem-solving achievement [61]. PE is defined as a personality trait characterised by a persistent investment of effort, interest and passion in long-term goals, particularly in the face of adversity [62,63]. When considered it in the context of learning mathematics, it manifests as mathematical perseverance, which refers to a student’s psychological and behavioural tendency to persist in problem-solving, engage in repeated practice, and maintain high levels of effort when confronted with difficult problems, errors, or failure [64–66]. From the perspective of goal setting theory (GST), PE is the fundamental safeguard that enables individuals to overcome obstacles and ultimately achieve long-term learning objectives [67–69]. Students with high levels of PE do not abandon their goals when faced with temporary setbacks, perceiving difficulties instead as transient problems to be solved. The sustained engagement not only directly enhances strategy proficiency, but also effectively mitigates negative emotions when facing challenges, thereby helping them maintain motivation for problem-solving and continuously refine their strategies within complex problem situations [70–72]. Such persistent investment significantly promotes academic achievement [62,73]. The prior research demonstrate that PE helps students maintain learning momentum and improve strategy fluency, as well as facilitates the accumulation of positive mastery experiences through successfully navigating challenges, thereby strengthening their SE [48,74]. More importantly, the role of PE may extend beyond direct promotion to acting as a crucial boundary condition that moderates the effects of other non-cognitive factors. The existing research provides important insights into the mechanisms of PE, and reveals several methodological and developmental relevance limitations. For example, Powell and Nettelbeck’s study found that, when fluid intelligence and conscientiousness were controlled for, general IC offered limited incremental predictive power for academic achievement. The result suggests that, in order for IC to be effectively translated into academic outcomes, it may require synergistic support from traits such as PE [33]. However, the previous study was limited to young adults aged 17–32 and utilized a cross-sectional design, preventing the examination of dynamic interactions among variables. By studying both high school and university students, Muenks et al. further deepened the understanding of PE’s structure, finding that although the “PE of effort” dimension of grit predicted grades, it was a less powerful predictor than other self-regulation variables [75]. Building on the evidence, the present study proposes a more refined theoretical model: within the pathway from IC to SE, PE likely functions as a booster [76]. Students who possess both strong IC and high levels of PE are more likely to transform their exploratory interest into sustained, in-depth engagement. In the process, the high-PE trait protects their SE from being undermined by temporary setbacks and supports their continued effort until problems are resolved, which ensures that the intrinsic motivation sparked by IC can be effectively translated into actual ability enhancement through the bridge of SE. The limitations of the existing literature in directly validating the moderation model, including constraints on the age groups of the sample and the research design, highlight the importance of using nationally representative, large-scale data on adolescents in the current research. Such data can be used to systematically examine the moderating role of PE within the “motivation-belief-ability” transmission pathway. Therefore, as a key personality trait, PE is suggested to be a crucial enhancing role in the complex mechanism through which students’ IC influences their MPSA.

Although the existing research has demonstrated the importance of non-cognitive factors on MPSA from motivational, cognitive belief, and personality trait perspectives, two critical questions remain inadequately addressed: how these factors interact synergistically, and for whom their mechanisms are most effective. The limitation constitutes a current research gap. Firstly, with regard to the integration of mechanisms, most studies focus on the independent effects of single variables or simple relationships between two variables, which means that they fail to empirically test the complete psychological pathway of “motivation (IC) → belief (SE) → trait (PE)” within a unified framework. Secondly, with regard to group heterogeneity, existing research largely assumes homogeneity among student populations and generally overlooks the potential moderating effects of key background variables, such as gender and ESCS. Students from different groups may exhibit systematic differences in the sources of their motivation, the ways in which they form beliefs, and their patterns of volitional engagement. Without examining these potential variations, the generalizability of research conclusions becomes questionable, and resulting educational interventions risk lacking specificity. Crucially, the Irish educational context offers a unique and valuable natural experiment with which to test these complex mechanisms. Despite having a highly standardized curriculum, Irish students exhibit a pronounced mathematics gap phenomenon. The contradiction strongly suggests that, in addition to uniform cognitive skill instruction, the mechanisms of non-cognitive factors and their group-specific variations may provide key insights into resolving this challenge. However, existing research lacks studies that simultaneously integrate multidimensional non-cognitive factors and systematically examine their group heterogeneity within the specific context. In order to address these dual gaps, the present study aims to construct a moderated mediation model. Using a sample of Irish adolescents, the research will systematically examine the mediating pathway through which IC influences MPSA via SE, as well as the moderating role of PE in the pathway. Furthermore, the current research will examine the universality and specificity of the theoretical model across different student groups through subgroup analyses based on gender and ESCS. Such exploration will provide robust empirical evidence for the development of differentiated and targeted mathematics education intervention strategies.

### The present study

To address these limitations, the present research integrates SDT and SCT to construct a moderated mediation model using a sample of Irish adolescents, which aims to systematically examine the mechanism through which IC influences MPSA, focusing specifically on the mediating role of SE and the moderating effect of PE. Accordingly, the current study proposes the following research questions: 1) How does IC directly influence adolescents’ MPSA? 2) Does SE mediate the relationship between IC and MPSA? 3) Does PE moderate the direct path from IC to MPSA and its indirect path via SE? 4) Do the aforementioned influence mechanisms differ significantly across student groups based on gender and ESCS?

**Fig 1:**
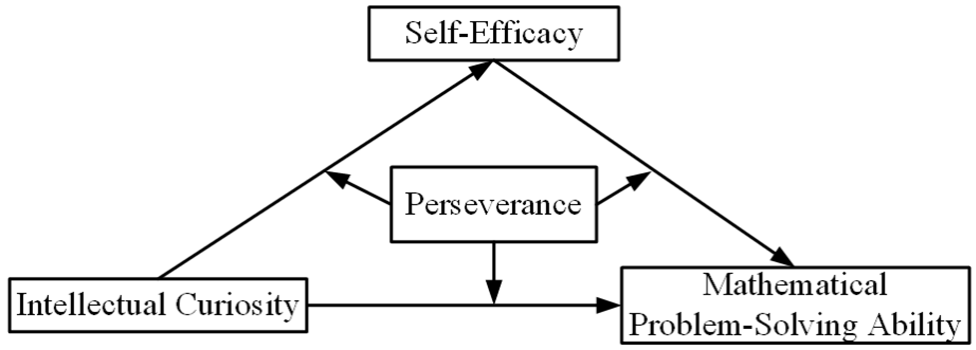
Theoretical moderated mediation model. The diagram illustrates the hypothesized relationships between intellectual curiosity (IC), self-efficacy (SE), perseverance (PE), and mathematical problem-solving ability (MPSA). Solid arrows represent direct effects: IC is hypothesized to directly influence MPSA (Path *c*; H1) and SE (Path *a*; H2a). SE is predicted to directly influence MPSA (Path *b*; H2b), constituting an indirect effect of IC on MPSA via SE (H2). Dashed lines from PE indicate its hypothesized moderating effects on three paths: (1) the relationship between IC and SE, (2) the direct relationship between IC and MPSA (Path $c^{\prime }$), and (3) the relationship between SE and MPSA (collectively, H3). Additionally, the model will be tested for heterogeneity across gender and economic, social, and cultural status (ESCS) groups through multigroup analysis (H4). All variables are conceptualized as latent constructs.

Based on the research questions, the following hypotheses are proposed. The first hypothesis (H1) posits that IC significantly promotes MPSA. The second hypothesis (H2) states that SE mediates the relationship between IC and MPSA. The third hypothesis (H3) proposes that PE moderates the pathways within the mediation mechanism of MPSA. The fourth hypothesis (H4) suggests that the complex mechanism influencing MPSA exhibits group heterogeneity. In summary, the current study tests the moderated mediation model in Figure 1 to elucidate how non-cognitive factors influence MPSA, including its underlying mechanisms and boundary conditions, thereby offering targeted insights for both theory and educational practice.

## Methods

### Participants

The current study utilizes data primarily derived from PISA 2022. PISA is an international educational assessment project conducted every three years since 2000 organized by OECD, with each cycle focusing on different domains. It aims to evaluate the comprehensive literacy of secondary school students worldwide in reading, mathematics, and science. PISA 2022 covered 81 countries and surveyed a total of 613,744 students. The Irish education system is characterized by its highly standardized Leaving Certificate examination [8]. However, Irish students showed a significant imbalance in their performance in the PISA international assessment. While they consistently achieved high scores in science and reading, which were well above the OECD average, their performance in mathematics was only average. The uneven performance across subjects highlights the particular value of investigating the mechanisms influencing MPSA within a standardized educational framework. Ireland thus presents a highly representative research case, offering a unique empirical context for understanding the interaction between a unified examination system and students’ non-cognitive factors. An in-depth investigation into the factors influencing Irish adolescents’ MPSA has practical significance not only locally, but also provides a cross-cultural reference for mathematics learning and teaching in similar educational systems.

To ensure high quality and representativeness of the analytical sample, the present research employed a complete case analysis approach, excluding cases with missing data on all core variables including IC, SE, and PE. The Little’s missing completely at random (MCAR) test confirmed that the data missing pattern was MCAR. After removing 48 invalid cases following further data cleaning, the final valid sample comprised 5,521 students from 170 schools. A total of 2,739 (49.6%) participants were females and the remaining 2,782 (50.4%) were males. The average age of the participants was 15.733 years (SD=0.294), and 99.3% of the students were in Grade 10, 11, or 12. It should be noted that PISA assessments use student age (15 years old) as the core sampling criterion rather than grade level, aiming to facilitate international comparisons across education systems [7]. In the final sample of the present study, 99.3% of students were in grades 10–12 (corresponding to the third year of junior high school to the second year of senior high school), while 0.7% were in other grades. The minimal grade heterogeneity is an inherent characteristic of PISA’s age-based sampling. Given its negligible proportion, its impact on overall analysis results is expected to be negligible. Retaining the entire sample maintains its representativeness of Ireland’s 15-year-old student population and aligns with PISA’s technical specifications for data analysis. Additionally, the index of ESCS is a core composite indicator derived from the PISA student questionnaire, designed to comprehensively measure students’ family socioeconomic background. The index is constructed based on three key dimensions: the highest occupational status of the parents, the highest level of parental education (in years), and home possessions, which serve as a proxy for family wealth. In the current study, the average ESCS index for Irish students was 0.347 (SD=0.801). As PISA sets the average for OECD countries at 0, the result indicates that the overall socioeconomic background of Irish students is significantly higher than the OECD average.

## Instruments

### Intellectual curiosity

IC was assessed using the PISA 2022 intellectual curiosity scale. The scale consists of 10 items, including statements such as “I am curious about many different things,” “I like asking questions,” “I like learning new things at school,” “I am more curious than most people I know,” “I find learning new things boring,” and “I enjoy learning new things.” All items were rated on a 5-point Likert scale ranging from “strongly disagree” (1) to “strongly agree” (5), with items IC3 and IC8 reverse-scored to align the direction of measurement. The OECD used the weighted likelihood estimation (WLE) method based on student responses to all ten items to construct the composite indicator for IC. The resulting scale was standardized such that the average for students in OECD countries was set to 0 with a standard deviation of 1, generating a standardized index named “CURIOAGR” to quantify students’ IC. To achieve superior measurement reliability, the scale was streamlined based on the results of confirmatory factor analysis. The five deleted items (IC2, IC3, IC7, IC8 and IC9) had standardized factor loadings of 0.42, 0.47, 0.45, 0.49 and 0.44 respectively, all of which were below the conventional threshold of 0.5. The final five-item scale demonstrated sound psychometric properties. Confirmatory factor analysis (CFA) revealed standardized factor loadings ranging from 0.628 to 0.808, all of which exceeded 0.5, indicating satisfactory construct validity [77]. Composite reliability (CR) was 0.837, with the average variance extracted (AVE) of 0.509, and Cronbach’s alpha was 0.830, indicating good internal consistency [78,79]. The square root of AVE exceeded the correlation coefficients between the variable and others, supporting the scale’s discriminant validity [80,81]. Furthermore, to rigorously assess the potential impact of scale revisions on research conclusions, the current study conducted a sensitivity analysis. The entire core model was re-examined using the official, unabridged 10-item PISA index. The results indicate that all key research inferences, including the significance, direction of effect, and group heterogeneity patterns of H1 to H4, remain stable regardless of whether they are based on the condensed 5-item index or the full 10-item index. Differences in core path coefficients did not exceed 5%. The result confirms the robustness of the findings with respect to the specific operationalization of IC. Given the simplified scale’s superior internal consistency and lack of impact on scientific inference, subsequent sections report analyses based on the five-item scale.

### Self-efficacy

Guided by the SCT of SE, the present study employed two complementary scales provided by PISA 2022 to measure students’ confidence in completing a series of mathematical tasks across different contexts. The two sub-dimensions focused on “formal and applied mathematics tasks” and “mathematical reasoning and 21st-century tasks” respectively. The former was assessed using 9 tasks, such as “calculating the price of a computer including tax”, while the latter was evaluated through 10 tasks, such as “extracting mathematical information from a chart”. All items were self-rated on a 4-point Likert scale ranging from “not at all confident” to “very confident”. Based on student responses to all 19 items, the OECD employed the WLE method to generate two separate composite indices named as “MATHEFF” and “MATHEF21”. The two indices were found to be highly correlated, allowing them to be merged into a single overall mathematical self-efficacy variable. Specifically, the scores from both dimensions were summed and averaged, with higher scores indicating higher levels of SE. The overall scale demonstrated acceptable internal consistency, with Cronbach’s α coefficient of 0.740 for the current study. The CFA results showed that the factor loadings for all items ranged from 0.720 to 0.835, all exceeding the threshold of 0.5, indicating good structural validity. The CR was 0.755, and the AVE was 0.608, both meeting acceptable standards. Furthermore, the square root of the AVE was greater than the correlations between the variables, providing further evidence of satisfactory convergent and discriminant validity for the scale.

### Perseverance

The present study employed the perseverance scale utilized in PISA 2022 to assess students’ persistence and level of effort when confronting challenges and difficulties. The scale consists of 10 items, encompassing behavioral statements such as “I keep working on tasks until they are finished”, “I put forth extra effort when the work becomes challenging”, and so on. All items were rated on a 5-point Likert scale ranging from “strongly disagree” (1) to “strongly agree” (5), with items PE4, PE6, PE7, and PE10 being reverse-scored. Based on student responses to all 10 items, the OECD employed the WLE method to construct a composite perseverance index named “PERSEVAGR”. During the reliability and validity testing process, items PE4, PE6, PE7, and PE10 were removed based on statistical results, resulting in a final scale with sound psychometric properties. In the current research, the scale demonstrated high internal consistency, with Cronbach’s α coefficient of 0.859. CFA results showed that the factor loadings for all items ranged from 0.571 to 0.840, all exceeding the threshold of 0.5, demonstrating good structural validity. Further analysis showed that the CR was 0.862 and the AVE was 0.514, both meeting acceptable standards. Additionally, the square root of the AVE was greater than the correlations between the variables, indicating satisfactory convergent and discriminant validity for the scale.

### Mathematical problem-solving ability

The assessment of students’ MPSA in the current study is based on the “plausible values” methodology employed in PISA 2022. According to the PISA 2022 Technical Report [82], the plausible values (PVs) are not direct scores from specific test items. Instead, they are generated through a multi-dimensional latent regression model constructed using item response theory, based on students’ response patterns across all mathematics test items. This model employs a normal approximation to estimate the posterior distribution of student ability, yielding latent ability indicators that have found widespread application [83,84]. MPSA is conceptualized across four core cognitive dimensions: expressing, employing, interpreting and evaluating, and reasoning. Ten PVs are provided for each dimension to comprehensively reflect the distribution of student abilities, scaled to a metric where the OECD country average is 500 with SD of 100. Adhering to PISA technical standards recommending the use of PVs from the same draw for cross-dimensional analysis, this study selected PV1MPFS, PV1MPEM, PV1MPIN, and PV1MPRE as the representative indicators for the four respective dimensions, ensuring data source consistency and result comparability.

### Data processing

PISA employs a two-stage stratified random sampling design: schools are first selected through stratification by school type, geographic location, and socioeconomic indicators, followed by the random selection of age-eligible students within sampled schools. Due to the “matrix sampling” design implemented by the OECD, where each student responds to only a subset of 5 items rather than the complete set, the variables examined in the study including IC, SE, and PE exhibited missing data rates ranging from 45% to 56%. To obtain unbiased parameter estimates, multiple imputation was employed to handle missing values [85–87]. Subsequently, in accordance with the two-stage unequal probability sampling design of PISA, all analytical results were weighted, and standard errors were estimated using the replicate samples method to correct for biases introduced by the complex sampling design.

### Data analysis

To systematically test the hypotheses proposed in the present study, a series of progressive statistical analysis methods were employed, all conducted using SPSS 26.0 and AMOS 26.0 software.

First, to explore the fundamental relationships among core variables (IC, SE, PE, MPSA) and group differences, the current research employed descriptive statistics, Pearson correlation analysis, and intergroup comparisons. For comparisons involving the binary gender variable, an independent samples t-test was used. For ESCS, the study divided it into four ordered groups based on quartiles (Lowest, Low, High, Highest) [13,88–91]. When conducting group comparisons, preliminary tests indicated that the data did not satisfy the assumption of homogeneity of variance. Therefore, the more robust Welch’s analysis of variance was employed, supplemented by Tamhane’s T2 test for post-hoc multiple comparisons to clearly reveal whether a “socioeconomic gradient effect” existed across variables [92–94].

Second, to test the mediation hypotheses (H1, H2), the present study constructed a structural equation model (SEM) using AMOS 26.0 software and estimated it via maximum likelihood estimation. Model fit was assessed through a series of indices: $\chi^{2}/df$, CFI, TLI, SRMR, and RMSEA. Generally, CFI and TLI > 0.90, along with RMSEA < 0.08, indicate good model fit [95]. The significance of mediating effects is tested using the Bootstrap method, with 5000 resamples and calculation of bias-corrected 95% confidence intervals (CI). If the CI does not include 0, the effect is considered significant.

Subsequently, to examine the moderating role of perseverance (H3), the study conducted a moderated mediation analysis using Model 59 from the PROCESS macro developed by Hayes [96–98]. The model allows simultaneous testing of the direct effect of the independent variable on the dependent variable, the mediating effect, and the moderating effect of the moderator variable on both the direct path and the first and second halves of the mediating path. The presence of a moderation effect was assessed by examining whether the interaction terms between IC and PE significantly predicted SE and MPSA, and whether the interaction term between SE and PE significantly predicted MPSA. Significant interaction effects will be further interpreted through simple slope analysis, examining the effects of IC on SE, IC on MPSA, and SE on MPSA at one standard deviation above and below the mean of PE.

Finally, to test the group heterogeneity hypothesis (H4), the current research conducted multi-group structural equation modeling based on the validated mediation model. Gender and ESCS were used as grouping variables. The analysis followed these steps: (1) Run the baseline model in each subgroup to ensure good model fit across groups; (2) Conduct measurement invariance tests by progressively constraining factor loadings and intercepts to be equal across groups, confirming the measurement tools hold the same meaning in different populations; (3) Conducted structural invariance tests by constraining structural path coefficients (IC → SE, SE → MPSA, IC → MPSA) to be equal across groups and comparing the chi-square difference between constrained and unconstrained models. A significant $\Delta \chi^{2}$ test indicated substantial group differences in path coefficients, confirming the presence of moderation effects [99,100].

## Results

### Descriptive statistics and correlation analysis

To further investigate the potential interrelationships among IC, SE, PE, and MPSA, the present study conducted descriptive statistics and Pearson correlation analyses on the composite indicators of these variables, as presented in Table 1. It should be noted that the overall average levels for IC, SE, and PE across OECD countries are set to 0, with SD of 1, whereas for MPSA, the average is set to 500 with SD of 100 [82]. The descriptive statistics reveal that the mean score for IC among Irish students was −0.002, essentially equivalent to the overall OECD average (*M* = 0). The SD was 0.887, slightly lower than the OECD average (SD = 1), indicating that the IC of the Irish students was generally stable with relatively small between-individual differences. However, Irish students’ performance in SE, PE, and MPSA were −0.087, −0.017, and 491.806, respectively, all below the corresponding OECD average levels. The finding suggests that Irish students may lack sufficient confidence and sustained motivation when facing challenges, and also have room for improvement in problem-solving ability. Additionally, the difference analysis using one-sample t-tests showed that the IC of Irish adolescents did not differ significantly from the overall OECD average, whereas significant differences (p< 0.05) were found for SE, PE, and MPSA. Furthermore, the correlation analysis results indicated significant positive correlations among IC, SE, PE, and MPSA. Although the strengths of the correlations varied, the psychological traits are interrelated with learning ability and collectively influence students’ learning engagement and problem-solving performance.

**Table 1:** The descriptive statistics and correlation analysis.

Variable	Min	Max	Mean	SD	1	2	3	4
1.IC	−4.020	4.050	−0.002	0.895	1			
2.SE	−2.950	2.580	−0.087	0.898	0.304**	1		
3.PE	−5.970	4.720	−0.017	0.907	0.336**	0.334**	1	
4.MPSA	232.040	760.020	491.806	80.950	0.268**	0.463**	0.222**	1

Note: IC = intellectual curiosity; SE = self-efficacy; PE = perseverance; MPSA = mathematical problem-solving ability; ** correlation is significant at the 0.01 level (two-tailed).

To systematically examine the influence of demographic variables on variables such as IC, the present study conducted independent samples t-tests. The results, presented in Table 2, reveal significant differences between male and female students in IC, SE, PE, and MPSA, with males outperforming females across all dimensions. Furthermore, Table 3 indicates that ESCS group comparisons further reveal a significant socioeconomic gradient effect. Specifically, as ESCS levels increase, students’ performance in IC, SE, PE, and MPSA shows a clear sequential upward trend, with the Highest ESCS group demonstrating the strongest performance. These findings suggest that Irish students’ psychological traits and academic abilities not only exhibit significant gender differences but are also systematically influenced by family socioeconomic and cultural status.

**Table 2:** The results of independent-samples *t* tests by gender.

Variable	Gender	*N*	Mean	SD	*t*	*p*	Comparison
IC	1	31,655	−0.028	0.872	−7.111***	<0.001	Male > Female
	2	33,069	0.022	0.916			
PE	1	31,655	−0.148	0.871	−36.546***	<0.001	Male > Female
	2	33,069	0.110	0.923			
SE	1	31,655	−0.214	0.851	−35.425***	<0.001	Male > Female
	2	33,069	0.034	0.925			
MPSA	1	31,655	485.311	76.519	−20.077***	<0.001	Male > Female
	2	33,069	498.024	84.510			

Note: 1 = female; 2 = male; IC = intellectual curiosity; PE = perseverance; SE = self-efficacy; MPSA = mathematical problem-solving ability; *** p< 0.001.

**Table 3:** The results of one-way ANOVA by ESCS.

Variable	ESCS level	*N*	Mean	SD	*F*	*p*	Tamhane
IC	1. Lowest ESCS	16 186	−0.189	0.848	602.051***	<0.001	4>3>2>1
	2. Low ESCS	16 183	−0.062	0.866			
	3. High ESCS	16 177	0.027	0.875			
	4. Highest ESCS	16 178	0.217	0.941			
SE	1. Lowest ESCS	16 186	−0.347	0.894	1213.087***	<0.001	4>3>2>1
	2. Low ESCS	16 183	−0.173	0.822			
	3. High ESCS	16 177	−0.052	0.869			
	4. Highest ESCS	16 178	0.223	0.908			
PE	1. Lowest ESCS	16 186	−0.169	0.915	361.174***	<0.001	4>3>2>1
	2. Low ESCS	16 183	−0.062	0.839			
	3. High ESCS	16 177	0.013	0.915			
	4. Highest ESCS	16 178	0.151	0.927			
MPSA	1. Lowest ESCS	16 186	454.986	75.095	3071.784***	<0.001	4>3>2>1
	2. Low ESCS	16 183	477.101	74.547			
	3. High ESCS	16 177	504.045	73.219			
	4. Highest ESCS	16 178	531.114	79.926			

Note: ***p<0.001, **p<0.01, *p<0.05.

### The mediating mechanism from intellectual curiosity to mathematical problem-solving ability

**Fig 2:**
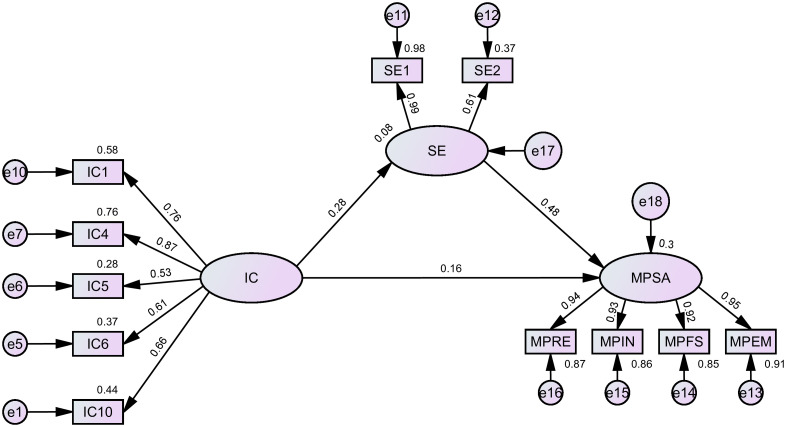
The Structural Equation Model of Mathematical Problem-Solving Ability. The structural equation model shows the direct and indirect effects among intellectual curiosity (IC), self-efficacy (SE), perseverance (PE), and mathematical problem-solving ability (MPSA). Standardized path coefficients are displayed, with all paths significant, supporting H1 and H2.

The current research employed structural equation modeling to examine the mediating effect of SE between IC and MPSA. The model demonstrated good fit: $\chi^{2}(40)=9714.474$, CFI=0.981, NFI=0.981, TLI=0.974, RMSEA=0.061, all meeting the recommended thresholds proposed by Hu and Bentler [95]. The analysis of standardized path coefficients (Table 4) indicates that IC significantly influences both students’ SE (β=0.280) and MPSA (β=0.160). Furthermore, SE itself significantly and positively affects MPSA (β=0.477). The 95% confidence intervals for all three paths excluded zero, confirming the presence of mediating effects. The present study further employed bias-corrected Bootstrap methods to test the significance of mediating effects, with results shown in Table 5 and Figure 2. The direct effect value of IC → MPSA was 0.160, accounting for 54.60% of the total effect; The indirect effect value of IC → SE → MPSA was 0.133, accounting for 45.40% of the total effect. Since the 95% confidence interval did not include zero, the result indicates that SE partially mediated the relationship between IC and MPSA. In summary, IC not only directly promotes MPSA, but also exerts an indirect effect through the mediation of SE, supporting H1 and H2.

**Table 4:** The relationships among intellectual curiosity, self-efficacy, perseverance, and mathematical problem-solving ability.

Path	Estimate	β	S.E.	*p*	Lower	Upper
IC → SE	0.589	0.280	0.009	***	0.270	0.290
SE → MPSA	36.509	0.477	0.424	***	0.469	0.485
IC → MPSA	25.849	0.160	0.669	***	0.152	0.169

Note: ***p<0.001, **p<0.01, *p<0.05.

**Table 5:** The standardized mediation effects of self-efficacy.

Path		Estimate	*p*	Lower	Upper	Effect Size
IC → SE → MPSA	Total effect	0.294	***	0.286	0.302	
	Direct effect	0.160	***	0.152	0.169	54.60%
	Indirect effect	0.133	***	0.128	0.139	45.40%

Note: ***p<0.001, **p<0.01, *p<0.05.

### Moderating effect of perseverance on the mediating role of self-efficacy

The results of testing the moderating effect of PE in the mediation model IC → SE → MPSA are presented in Table 6. It has been suggested that both IC (β=0.221) and PE (β=0.279) significantly and positively predict SE. Furthermore, IC (β=0.134), SE (β=0.408), and PE (β=0.279) all significantly and positively predict MPSA. PE (β=0.279) significantly and positively predict SE. Furthermore, IC (β=0.134), SE (β=0.408), and PE (β=0.055) all exert significant direct predictive effects on MPSA. More crucially, the interaction term between IC and PE (int1) significantly predicted not only SE (β=−0.043) but also MPSA (β=−0.045). Furthermore, the interaction term between SE and PE (int2) significantly predicted MPSA (β=0.036). The result indicates that PE plays a moderated mediating role throughout the mediation pathway. It moderates both the mediating process of SE between IC and MPSA, and directly moderates the direct path from IC to MPSA as well as the influence path from SE to MPSA.

**Table 6:** The moderated mediation analysis of perseverance.

Predictor	SE as dependent variable				MPSA as dependent variable			
	β	*p*	Lower	Upper	β	*p*	Lower	Upper
Constant	0.015	***	0.007	0.022	0.003	0.381	−0.004	0.010
IC	0.221	***	0.214	0.229	0.134	***	0.126	0.141
SE					0.408		0.400	0.415
PE	0.279		0.272	0.287	0.055		0.048	0.063
Int1	–0.043		–0.048	0.038	0.045		–0.050	–0.039
Int2					0.036		0.030	0.042
$R^{2}$	0.157				0.237			
*F*	4008.456***				4020.778***			

Note: Int1 = IC × PE, Int2 = SE × PE; ***p<0.001, **p<0.01, *p<0.05.

**Fig 3:**
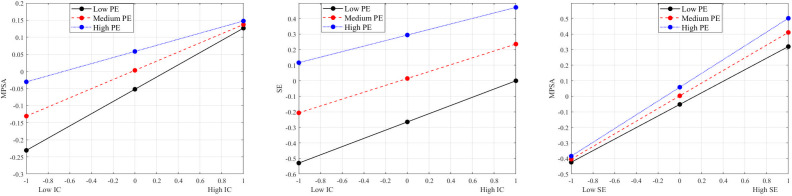
The analysis results of simple slope plot. The moderating effects of PE on three key paths (H3). PE negatively moderates the effect of IC on SE; PE negatively moderates the direct effect of IC on MPSA; PE positively moderates the effect of SE on MPSA. These patterns reveal PE’s dual role of “inhibiting input and amplifying output” in the motivational process. Additionally, cross-group comparisons (H4) indicated significant heterogeneity in the mediating mechanism across gender and ESCS groups.

To clarify the direction of PE’s moderating effects, simple-slope tests were performed and plotted in Figure 3. When PE was low, the regression slopes of IC → SE (β=0.265, p<0.001) and IC → MPSA (β=0.179, p<0.001) were steep. As PE increased, both slopes flattened (high PE: $\beta_{\text{IC-SE}}=$0.178, $\beta_{\text{IC-MPSA}}=$0.089, p<0.001). Thus, higher PE attenuates the positive predictive power of IC on SE and MPSA, indicating a negative moderating role along these two paths. Conversely, PE amplified the SE→MPSA link ($\beta_{\text{low}}=$0.372, $\beta_{\text{high}}=$0.443, p<0.001), reflecting a positive moderating effect. In summary, PE exhibits significant moderating effects on both the direct path from IC to MPSA and the intermediary path from IC to SE to MPSA, specifically on the first segment (IC → SE) and the second segment (SE → MPSA).

Further analysis indicates that, at low, medium and high levels, the β values for the predictive coefficient of IC on MPSA are 0.179, 0.134 and 0.089 respectively. The mediation effect values for SE are 0.098, 0.090 and 0.079 respectively. The corresponding 95% CI for all mediators do not include zero, confirming the statistical significance of all mediating effects. 0.089, respectively. The mediation effect values for SE were 0.098, 0.090 and 0.079 respectively, with the corresponding 95% CI not containing zero. The result indicates significant mediation effects. However, the effect strength decreased as PE increased. Furthermore, the 95% CI for the moderated mediation effect indices did not include zero, indicating significant differences in the mediating effect of SE across different levels of PE, thus confirming the existence of moderated mediation. In summary, PE exerts its influence through the dual mechanisms of “inhibiting input” and “amplifying output”, thereby validating H3.

### Multi-group analysis of the mechanism underlying mathematical problem-solving ability

To systematically clarify the robustness of the complex mechanism “IC → SE → MPSA” across different demographic characteristics, this part conducted multi-group structural equation modeling by gender and ESCS, and accordingly propose differentiated improvement strategies.

### Gender-based multi-group comparison

The multi-group structural equation modeling analysis revealed that both the male and female models exhibited excellent overall fit indices. The models yielded the following values: $\chi^{2}(40)=5115.289$, GFI=0.973, CFI=0.981, TLI=0.974, RMSEA=0.062 for males; $\chi^{2}(40)=5736.418$, GFI=0.969, CFI=0.977, TLI=0.968, RMSEA=0.067 for females. However, measurement and structural invariance tests indicate that the theoretical model does not exhibit full invariance across gender groups (p< 0.001). The result means that the influence mechanism of IC and SE on MPSA differs between genders.

Critical ratio (CR) analysis revealed no significant gender difference in the path from SE to MPSA (CR = 1.075). However, significant gender differences existed in the paths from IC to SE (CR=−8.384) and from IC to MPSA (CR=−2.777). The mediation analyses further as seen in Table 7 revealed that, within both gender groups, IC exerted an indirect influence on MPSA via SE, forming a significant mediating pathway. However, the indirect effect accounted for 45.60% in the male group, significantly higher than the 43.30% in the female group, indicating that SE plays a more crucial mediating role in the cognitive mechanisms of males. In summary, gender moderates the influence mechanism of IC → SE → MPSA, thereby supporting H4.

**Table 7:** The gender subgroup analysis of mediation effects of self-efficacy.

	Path	Estimate	*p*	Lower	Upper	Effect %
Male	Total effect	0.312	***	0.301	0.323	
	Direct effect	0.170	***	0.158	0.182	54.40%
	Indirect effect	0.142	***	0.134	0.151	45.60%
Female	Total effect	0.270	***	0.258	0.282	
	Direct effect	0.153	***	0.141	0.165	56.70%
	Indirect effect	0.117	***	0.109	0.125	43.30%

Note: ***p<0.001, **p<0.01, *p<0.05.

### Multi-group comparison based on ESCS

The multi-group structural equation modeling analysis revealed that all ESCS group models demonstrated satisfactory overall fit indices. Overall fit was satisfactory for all ESCS groups: $\chi^{2}(40)=2506.788$, GFI=0.973, CFI=0.979, TLI=0.971, RMSEA=0.062 for Lowest ESCS; $\chi^{2}(40)=2493.783$, GFI=0.973, CFI=0.979, TLI=0.972, RMSEA=0.062 for Low ESCS; $\chi^{2}(40)=2669.364$, GFI=0.970, CFI=0.977, TLI=0.969, RMSEA=0.064 for High ESCS; $\chi^{2}(40)=3448.351$, GFI=0.939, CFI=0.974, TLI=0.964, RMSEA=0.073 for Highest ESCS, indicating good model fit across different gender groups. Further measurement and invariance tests revealed that the theoretical model did not exhibit full invariance across ESCS groups (p< 0.001), demonstrating that the influence mechanism of IC and SE on MPSA exhibits structural differences across socioeconomic status groups.

The CR analysis of specific pathways reveals significant differences across ESCS groups in terms of key pathways. For instance, compared to the Lowest ESCS group, the Low ESCS group exhibited significant differences on the “IC → SE” and “IC → MPSA” pathways (CR=−4.289 and −2.835, respectively), while the High ESCS group showed a significant difference on the “SE → MPSA” (CR=−4.116). The mediation analyses, as depicted in Table 8, further revealed that, although IC consistently influenced MPSA indirectly through SE across all ESCS quartiles, the strength of the indirect effect varied in a graded fashion. Notably, the total effects were relatively strongest at the lowest (0.314) and highest (0.277) ESCS levels. Specifically, in the low ESCS group, the direct effect accounted for 62.10% of the total effect, significantly higher than other levels, indicating the group relies more heavily on the direct drive pathway of IC.

**Table 8:** The subgroup analysis of mediation effects of self-efficacy by ESCS.

	Path	Estimate	*p*	Lower	Upper	Effect Size
Lowest ESCS	Total effect	0.314	***	0.299	0.329	
	Direct effect	0.171	***	0.154	0.189	54.60%
	Indirect effect	0.143	***	0.131	0.154	45.40%
Low ESCS	Total effect	0.269	***	0.252	0.285	
	Direct effect	0.167	***	0.149	0.185	62.10%
	Indirect effect	0.102	***	0.091	0.113	37.90%
High ESCS	Total effect	0.217	***	0.200	0.235	
	Direct effect	0.118	***	0.107	0.130	54.20%
	Indirect effect	0.099	***	0.083	0.116	45.80%
Highest ESCS	Total effect	0.277	***	0.261	0.293	
	Direct effect	0.156	***	0.140	0.173	56.40%
	Indirect effect	0.121	***	0.108	0.133	43.60%

Note: ***p<0.001, **p<0.01, *p<0.05.

In summary, ESCS not only moderates the overall effect strength of IC on MPSA, but also shapes two distinct heterogeneous pathways: “direct drive” and “efficacy mediation”, further supporting H4.

## Discussion

Drawing on a nationally representative sample of Irish adolescents, the current study systematically examined how IC influences MPSA by uncovering the mediating role of SE and the moderating function of PE. Addressing the four core research questions, the present research obtained four valuable sets of findings.

### Direct effect of intellectual curiosity on mathematical problem-solving ability: the core role of intrinsic motivation

The foremost finding of the present study is that IC exerts a significant direct positive effect on adolescents’ MPSA. The result underscores the fundamental status of intrinsic motivation in the development of complex cognitive competence and can be coherently interpreted within classical motivational frameworks. From the perspective of expectancy value theory (EVT), highly curious students naturally ascribe greater intrinsic value and interest to mathematical activities [101,102]. They regard mathematical inquiry as an intellectually attractive adventure rather than a mere means to external ends. Such positive intrinsic valuation directly propels students to invest more frequently and deeply in mathematical problem-solving processes, thereby furnishing a sustained motivational basis for ability development [103]. A deeper theoretical account is provided by SDT, which posits that curiosity, as a pure form of intrinsic motivation, originates from individuals’ inherent pursuit of autonomy and competence [104]. In the process of learning mathematics, curiosity drives students to explore independently, satisfying their need for autonomy. They experience intense feelings of mastery and accomplishment when they understand a concept or solve a problem, which satisfies their need for competence. The self-initiated and self-rewarding learning process not only makes the activity itself reinforcing, but also promotes strategic experimentation and cognitive engagement during the process of problem solving, producing a direct enhancement of MPSA.

Additionally, cognitive neuroscience provides biological evidence for the direct motivation-to-ability link. The arousal of IC is closely associated with activation of the brain’s reward circuitry, particularly the mesolimbic dopaminergic pathway [105]. When adolescents confront novel or uncertain mathematical problems, dopaminergic firing not only encodes prediction errors, but also amplifies the intrinsic pleasure and motivational salience of exploratory behavior [106]. The positive neuro-modulatory state enhances attentional focus, cognitive flexibility and overall problem-solving performance [107,108]. Taken together, the findings show that curiosity is not just a personality trait among Irish adolescents, but an intrinsic driver of MPSA. By satisfying basic psychological needs and recruiting associated reward mechanisms, curiosity supplies indispensable motivational fuel for complex cognitive activity.

### The mediating role of self-efficacy from motivation to ability

The present study further elucidates the underlying mechanism through which IC influences MPSA, SE functions as a key mediator. The finding indicates that IC not only directly fosters mathematical competence, but also indirectly enhances problem-solving performance by shaping students’ beliefs about their own mathematical capabilities. From the perspective of SCT, the mediating effect receives robust theoretical support [31]. Bandura posits that SE is primarily formed through four sources of information, among which mastery experience is the most influential [48]. In the context of the current study, a strong desire for knowledge is likely to serve as a vital driving force for creating mastery experiences. It drives students to engage continuously in mathematical exploration, accumulating successful experiences in the process of solving authentic problems. These successes, achieved through curiosity-driven efforts, help gradually build students’ firm belief that “I can do it” [74], thereby establishing a crucial psychological bridge between their thirst for knowledge and their mathematical abilities.

At the neural level, the facilitative effect of SE is presumably linked to functional optimization of the prefrontal cortex. As the core hub responsible for executive functions, including working memory and cognitive control [109], the prefrontal cortex exhibits activity patterns that covary significantly with perceived SE. Neuroimaging evidence indicates that individuals with high self-efficacy display more efficient and coordinated activation in the dorsolateral prefrontal cortex when confronting challenging tasks [110]. The optimized pattern of neural activity may enable individuals to allocate attentional resources more efficiently, flexibly employ problem-solving strategies, and suppress distracting information, thereby helping to translate cognitive potential into practical problem-solving abilities [111]. Functional near-infrared spectroscopy further shows a positive correlation between students’ SE levels and dorsolateral prefrontal activation while they engage in mathematical problem-solving tasks. Collectively, these findings suggest that SE serves as a “neural bridge” connecting motivational drive to cognitive output by tuning the functionality of key control regions [112,113]. Uncovering the mediating mechanism carries important instructional implications. As Pajares remarked, belief systems are the obligatory passage through which motivation is converted into achievement [114]. The educational interventions that stimulate curiosity while neglecting SE are likely to yield diminished returns. Consequently, mathematics instruction should systematically cultivate students’ SE via appropriately challenging tasks, mastery experiences and targeted feedback while simultaneously fostering IC, thereby more effectively promoting the development of MPSA. It should be noted, however, that SE must be understood within its broader socio-cultural context. Although the present study confirms its core mediating role, practitioners need to recognize that self-reported “high confidence” is context-sensitive. Regardless of the cultural context or classroom environment, responses to questionnaires may be influenced by social desirability. Moreover, when perceived confidence is misaligned with actual competence, it may be interpreted as unconstructive arrogance. Adopting a dialectical perspective implies that the ultimate goal of educational intervention is not just to increase self-efficacy scores, but also to establish students’ belief in their own capabilities on the basis of solid knowledge acquisition and sustained successful problem-solving experiences. The approach achieves an integrated and coherent advancement of both confidence and competence.

### Dual Moderating Role of Perseverance: A Resource-Allocation Mechanism of “Suppressing Input” and “Amplifying Output”

Employing a moderated mediation framework, the present study revealed that PE exerts a unique dual moderating effect on the psychological pathway linking IC to MPSA. Specifically, PE operates in a “suppress input, amplify output” manner: it negatively moderates the effect of IC on SE and on MPSA, while positively moderating the impact of SE on MPSA. The finding uncovers the complex function of PE in cognitive resource optimization and transcends the traditional view that simply treats PE as a linear facilitator [73]. From the perspective of Goal Pursuit Theory (GPT), the dual regulatory role of PE may reflect its distinct functions in motivation conversion and belief implementation. During the process of converting motivation into self-efficacy, high levels of perseverance may require more cognitive resources. However, excessive persistence may inhibit immediate motivation conversion by occupying these cognitive resources. The pattern is consistent with the finding of Wolters and Hussain that excessive persistence can undermine performance by precipitating cognitive overload [67]. Likewise, DiNapoli noted that rigid persistence without strategic adjustment may become maladaptive [63].

On the other hand, in the process of transforming beliefs into performance, high PE can enhance the positive predictive role of SE in problem-solving ability. When individuals have already established a certain level of self-efficacy belief, high PE may help maintain high levels of effort and commitment when facing complex problem-solving processes and setbacks. The “amplify output” effect is consistent with the secondary control postulate of control value theory (CVT) [115], which posits that persistence optimizes the conversion of existing resources into outcomes. The cross-cultural evidence supplied by Xu et al. corroborates that PE strengthens the positive relation between learning motivation and academic achievement [76]. From the perspective of the self-regulated learning framework, the dual-regulation mechanism may reflect the distinctive allocation of cognitive resources among highly persevering individuals. They likely achieve a balance between regulating motivational inputs (manifested as inhibition) and amplifying belief outputs (manifested as amplification), thereby more effectively translating motivation into actual competence. The sequence aligns with Metcalfe and Mischel’s (1999) hot/cool-system account, which posits that effective self-regulation hinges on inhibiting immediate impulses while maintaining sustained commitment to long-term goals [116]. Therefore, the current study provides a more nuanced view of the role of PE: by regulating both the conversion efficiency of motivational inputs and belief outputs simultaneously, it may achieve optimal allocation of limited mental resources across different cognitive processes. Pedagogically, the results caution against indiscriminately urging students to “persist more”. Instead, educators should cultivate “wise persistence” that couples curiosity with metacognitive skills for dynamically adjusting effort strategies according to task demands and personal states, thereby maximizing the adaptive benefits of PE.

### Cross-group heterogeneity in the mechanism underlying mathematical problem-solving ability

Multi-group analyses revealed that the mediating pathway “IC → SE → MPSA” exhibits systematic variation across gender and ESCS, underscoring the context-dependent nature of non-cognitive influences on mathematical competence. Within Ireland’s educational context, which is characterised by comparatively weak mathematics performance, this finding carries important implications. Along the gender dimension, the mediation model displays pronounced differential patterns. The indirect effect of SE accounts for a significantly larger proportion of the total effect among males than among females, a discrepancy likely rooted in distinct gender socialization processes within Irish schools. From a gender socialization theory perspective, males are often expected to perform better in mathematics and are given more opportunities to practice. The phenomenon might make them more likely to quickly turn their experiences from being curious and intellectual into a strong belief in what they can do [117]. In contrast, although females benefit from the same mediating channel, their SE formation may be complicated by additional factors such as stereotype threat [118].

On the ESCS dimension, a clear gradient emerged, which was evident in the data. Students from low-ESCS backgrounds exhibited a markedly higher direct-effect ratio, indicating that IC influences MPSA primarily through the direct path rather than via SE. The pattern aligns with the resource-scarcity framework [119]. When familial support is limited, learners tend to adopt more immediate learning strategies, rapidly converting curiosity into concrete behaviours instead of relying on the psychologically resource-intensive mediation of SE. While the strategy reflects adaptive learning under constraint, it also signals a relatively under-developed SE pathway. Conversely, high-ESCS students displayed a complete “motivation → belief → ability” transformation chain. Abundant family resources may provide them with more stable psychological and social support, which could enable IC to more smoothly influence mathematical ability through the mediating effect of SE [120]. The result mirrors documented ESES-related performance gaps within the Irish education system and highlights the pivotal role of family resources in shaping learning-psychological mechanisms. Based on the above findings, Irish educational interventions should adopt targeted strategies to reinforce the stability of STEM beliefs among males, while supporting multiple pathways for females to progress from curiosity to competence. Such differentiated approaches would enable Ireland to maintain its traditional subject strengths while effectively raising students’ MPSA and promoting overall educational quality.

Based on the above discussion, the following targeted educational intervention recommendations are proposed. Firstly, at the instructional design level, tasks involving a series of increasingly challenging steps, coupled with timely success feedback, should be implemented to strengthen the IC-to-SE conversion route. Secondly, in light of the dual nature of PE, differentiated training strategies are required. High-PE students should be guided to optimize their effort allocation, while low-PE students require structured support. Thirdly, interventions tailored to specific groups should be implemented. These include efficacy-reinforcement activities for males, multiple channels for displaying ability for females, enhanced provision of resources and immediate feedback for students from low-ESCS backgrounds, and guidance on in-depth inquiry for students from high-ESCS backgrounds. Finally, Ireland should implement mechanism-driven support systems. Teacher professional development should focus on mastering and applying the “motivation → belief → ability” conversion process, while home–school partnerships should deliberately cultivate a psychologically supportive climate that enables the joint development of all resources.

Despite offering valuable insights, the present study is subject to several limitations. Firstly, although all hypotheses are based on robust theory, the cross-sectional design prevents causal inference. As a result, the findings reflect associations rather than causal pathways. To determine the order of causation among IC, SE and MPSA, future work should use cross-lagged panel models or longitudinal tracking designs. Secondly, the relevant conclusions drawn from a single cultural context for Irish adolescents should be generalized with caution to other educational systems. Subsequent studies could use PISA multinational databases to make cross-cultural comparisons and examine how the proposed model varies in different cultural settings. Finally, self-report data are susceptible to common-method bias. To capture the dynamic interplay between non-cognitive factors and learning processes more objectively, future research should integrate complementary methodologies such as classroom observations, behavioral analytic and teacher ratings. Additionally, the current study sample included a very small proportion (0.7%) of 15-year-old students who were not enrolled in years 11–13. While the proportion is extremely low, and the PISA design is age-based, future research could validate the robustness of the model further using a sample composed entirely of students from the same year group.

## Conclusion

The present study, based on Irish PISA 2022 data, systematically reveals the synergistic mechanism through which IC, SE and PE influence adolescents’ MPSA, using a moderated mediation model. The results show that IC directly predicts MPSA and exerts a significant mediating effect through SE. Further findings reveal that PE plays a unique dual moderating role in this mechanism, exhibiting an “inhibitory input, amplifying output” pattern. Furthermore, the influence mechanism exhibits significant heterogeneity across gender and ESCS dimensions.

The current research advances the theoretical, methodological and practical dimensions. In terms of theory, it integrates SDT and SCT in order to construct and validate the motivational pathway of IC → SE → MPSA along with its boundary conditions. Methodologically, it leverages large-scale, cross-national assessment data (N = 5,521) and employs multigroup analysis to reveal group heterogeneity in the influencing mechanism. The method addresses limitations in the previous research that did not sufficiently account for individual differences. In practice, the present study revealed the intricate moderating role of PE, offering fresh insights into overcoming the linear mindset of “more perseverance is always better” and facilitating personalized teaching. Future research could employ longitudinal designs to further examine the causal relationships between the variables and validate the universality of the model across different cultural contexts.
